# Rare Case of a Clival Chondroma Resulting in Panhypopituitarism

**DOI:** 10.7759/cureus.49110

**Published:** 2023-11-20

**Authors:** Ekrem Yetiskul, Yisroel Y Grabie, Amira Hassan, Fatema Arafa, Salman Khan

**Affiliations:** 1 Department of Internal Medicine, Northwell Health - Staten Island University Hospital, Staten Island, USA

**Keywords:** benign tumors, cartilaginous tumour, altered mental status, panhypopituitarism, clival chondroma

## Abstract

Chondromas are rare benign tumors composed of hyaline cartilage that can arise in various locations in the body. Their occurrence in the clivus, leading to panhypopituitarism, is exceptionally rare. This case report describes a 93-year-old female with a known clival chondroma who presented with altered mental status, presumed to be secondary to toxic metabolic encephalopathy due to an infectious cause. Further diagnostic evaluation revealed pituitary hormone levels below the normal range. This case report aims to highlight a unique case of panhypopituitarism attributed to a chondroma in the clivus with tumor extension to the sellar region, emphasizing the diagnostic challenges and treatment options for this unusual pathology.

## Introduction

The clivus is a midline anatomical structure in the central skull base. It is affected by a wide range of non-neoplastic, benign, and malignant pathologies, some of which typically affect the clivus because of its strategic location and embryological origins [1]. Clival chondromas are rare, benign tumors characterized by the growth of hyaline cartilage in the clivus. These tumors pose unique diagnostic and management challenges due to their infrequent occurrence and proximity to vital neurovascular structures. Clival chondromas often remain clinically silent until they reach a size or stage that triggers significant complications, necessitating medical intervention. Complications are transformation to chondrosarcoma and increased risk of glial tumors. Bone destruction and soft tissue extension indicate malignant transformation [2]. One more potential complication of a clival chondroma is hypopituitarism, which is thought to be an uncommon condition with a prevalence of ~46 per 100,000 [3]. It can be due to a variety of tumoral, inflammatory, or infiltrative lesions or trauma or radiation of the hypothalamic-pituitary region, with the most common cause represented by non-functioning pituitary macroadenomas [4]. As a result, understanding the clinical presentation, diagnostic intricacies, and treatment approaches for clival chondromas is of paramount importance. In this case report, we present a patient who experienced the complication of panhypopituitarism attributed to a clival chondroma with tumor extension into the sellar region.

## Case presentation

Our patient, a 93-year-old female, has an extensive medical history that includes clival chondroma, renal cell carcinoma of the right kidney, chronic kidney disease (CKD) stage 4, hypertension, dyslipidemia, and recurrent urinary tract infections (UTI). She was brought to the emergency department by her daughter due to altered mental status. The daughter reported that she was taking ciprofloxacin for a UTI. Regrettably, the patient did not complete the entire course, as she had refused the final two days of treatment. The family noticed confusion and lethargy upon waking two days before admission, along with diffuse myalgias and abdominal pain. This behavior was a stark departure from her usual baseline mental status, where she was typically alert and oriented to person, place, and time.

Upon admission, the patient exhibited a blood pressure of 78/46 mm/Hg, a heart rate of 54 beats per minute, a fever with a maximum temperature of 100.6°F, and comfortable oxygen saturation on room air, registering 98% on the pulse oximeter. Despite appearing frail, she was not acutely distressed. Neurological examination revealed lethargy, with no gross deficits in cranial nerve function. There was a reduction in motor strength and muscle tone, and her coordination was compromised, but there were no focal neurological deficits. Cardiovascular and respiratory examinations did not reveal any abnormalities, and the abdominal examination showed no tenderness or masses, with normal bowel sounds.

**Figure 1:**
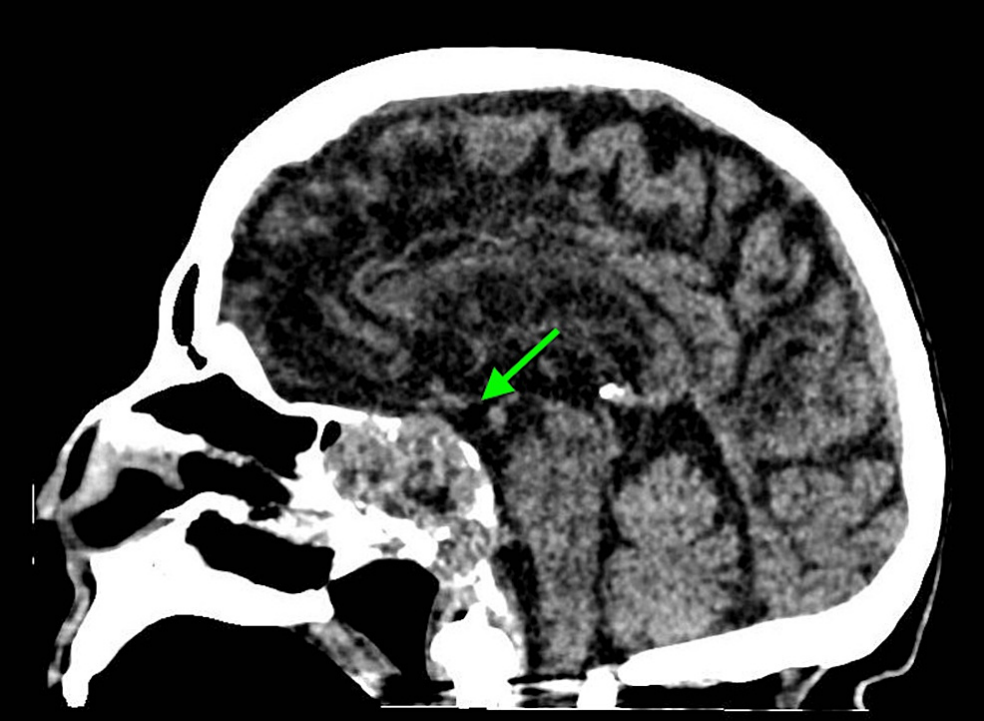
Sagittal image obtained from computed tomography of the head demonstrates clival chordoma with sphenoid sinus obliteration, involvement of the cavernous sinus, and bilateral petrous apices (green arrow)

**Figure 2:**
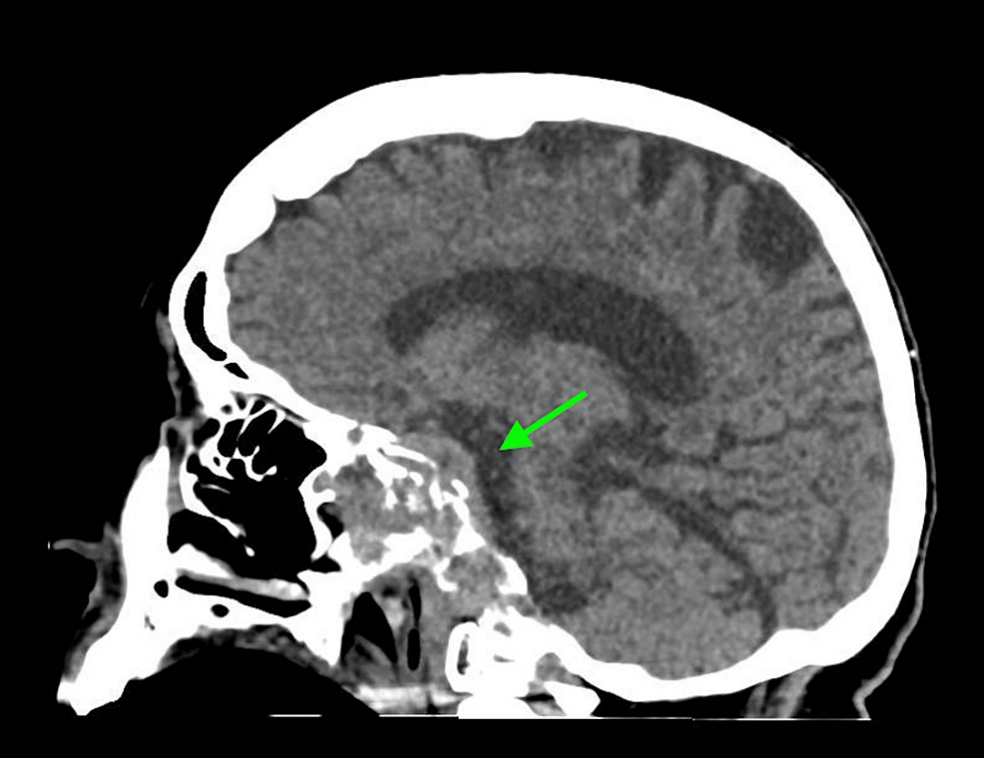
Sagittal image obtained from computed tomography of the head demonstrates clival chordoma with sphenoid sinus obliteration, involvement of the cavernous sinus, and bilateral petrous apices (green arrow)

**Figure 3:**
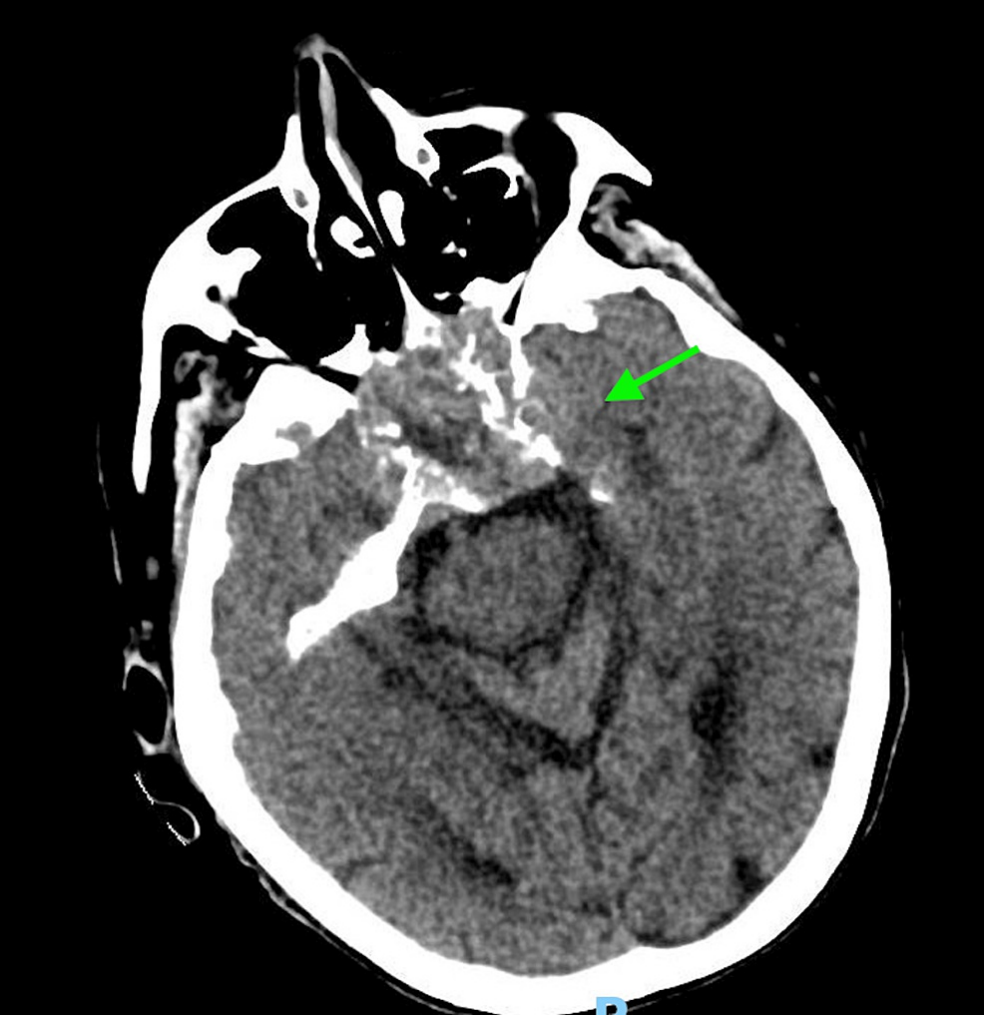
Axial image obtained from computed tomography of the head demonstrates clival chordoma with sphenoid sinus obliteration, involvement of the cavernous sinus, and bilateral petrous apices (green arrow)

**Figure 4:**
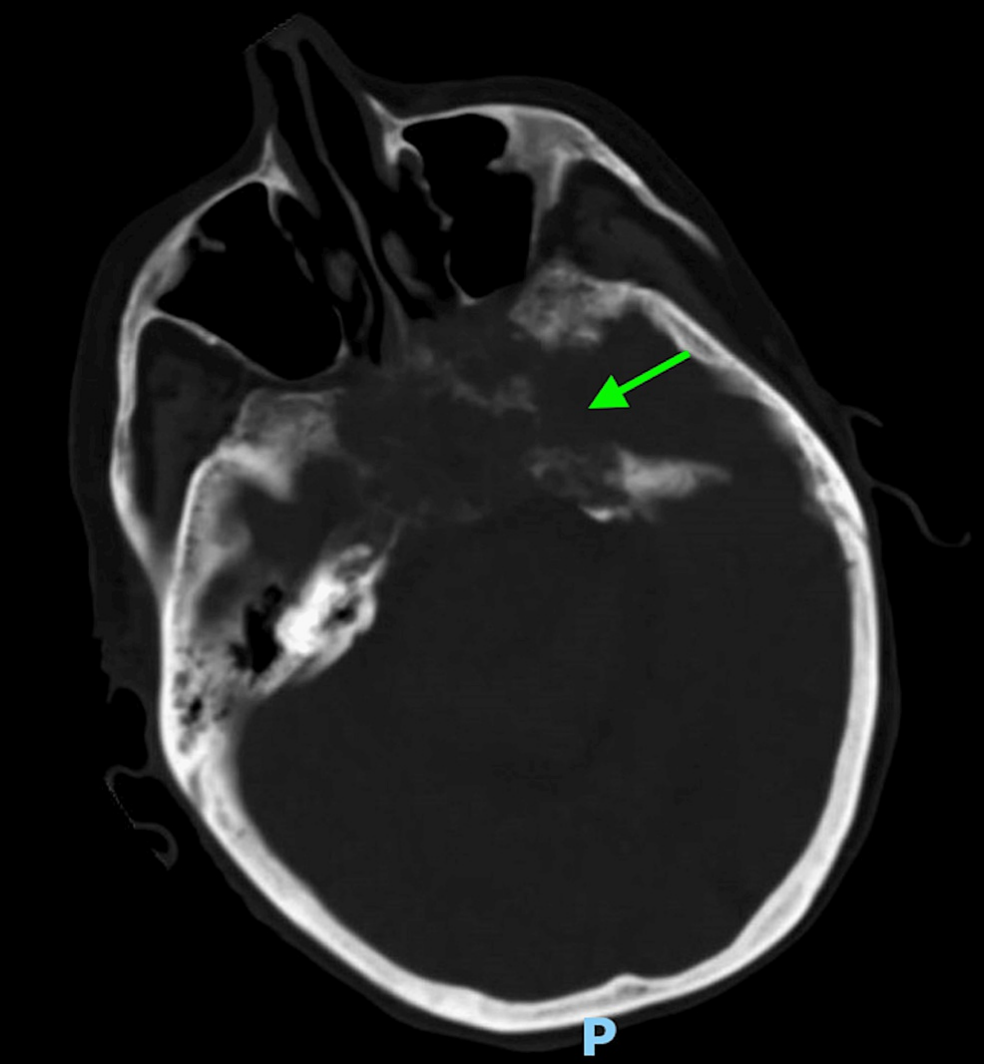
Axial image obtained from computed tomography of the head demonstrates clival chordoma with sphenoid sinus obliteration, involvement of the cavernous sinus, and bilateral petrous apices (green arrow)

Laboratory investigations upon admission revealed several significant findings, including a white blood cell (WBC) count of 10.9 K/uL (normal range: 4.80-10.80 K/uL), serum glucose of 57 mg/dL (normal range: 70-99 mg/dL), sodium of 132 mmol/L (normal range: 135-146 mmol/L), potassium of 5.8 mmol/L (normal range: 3.5-5.0 mmol/L), chloride of 100 mmol/L (normal range: 98-110 mmol/L), creatinine of 3.4 mg/dL (normal range: 0/7-1/5 mg/dL), blood urea nitrogen of 68 mg/dL (normal range: 10-20 mg/dL), and estimated glomerular giltration rate of 12 ml/min/1.73m² (normal range: >=60 mL/min/1.73 m²). Venous blood gas analysis revealed a pH of 7.26 (normal range: 7.32-7.43), partial pressure of carbon dioxide (pCO2) of 39 mmHg (normal range: 39-42 mmHg), and bicarbonate (HCO3) of 18 mmol/L (normal range: 22-29 mmol/L). Urinalysis and respiratory viral panel results were unremarkable. Blood and urine cultures were collected in the emergency department. A CT scan of the head showed an increased size of the clival chordoma with sphenoid sinus obliteration, involvement of the cavernous sinus, bilateral petrous apices, as well as a new left mastoid effusion, and left middle ear effusion (Figures 1-4). A CT of the abdomen and pelvis indicated an increase in the size of the known right upper pole clear cell renal cell carcinoma.

Given the patient's clinical presentation and past medical history, it was initially thought the patient presented with a UTI even though urinalysis was unremarkable. The patient was also noted to have an acute kidney injury, given her elevation from baseline creatinine, and we attributed her metabolic derangements, including hyponatremia, hyperkalemia, and metabolic acidosis, to renal impairment. In response to her condition, the patient received immediate treatment, which included a two-liter bolus of lactated Ringer's solution, intravenous piperacillin/tazobactam, and sodium bicarbonate tablets. Norepinephrine and lactated Ringer infusions were initiated, and she was admitted to the step-down critical care unit with a diagnosis of toxic metabolic encephalopathy, likely due to a combination of shock and uremia. A nephrology consult was requested for potential hemodialysis to address her altered mentation, presumably from uremia. However, the nephrologist doubted that uremia was the sole cause of her mentation. A recommendation to begin renal replacement therapy was made but the family declined to consent for this. Subsequent infectious evaluations did not pinpoint the source of the suspected sepsis, leading to a shift in focus to alternative potential causes.

Adrenal insufficiency was considered as a possible factor contributing to her symptoms. A morning cortisol test yielded a result of 6.0 ug/dL (normal range 6.0 - 18.4 ug/dL). Given her history of clival chondroma, radiological evidence of tumor expansion and destruction, current hypotension, hypoglycemia, and electrolyte imbalances, the possibility of panhypopituitarism was explored. Subsequently, various pituitary hormone levels were assessed, revealing a thyroid-stimulating hormone (TSH) of 0.26 uIU/mL (normal range: 0.27 - 4.20 uIU/mL), total thyroxine (T4) level of 4.7 ug/dL (normal range: 4.6 - 12 ug/dL), triiodothyronine (T3) level of 53 ug/dL (normal range: 80 - 200 ug/dL), corticotropin (adrenocorticotropic hormone (ACTH)) level of 4.9 pg/mL (normal range: 7.2 - 63.3 pg/mL), and prolactin (PRL) level of 133.0 ng/mL (normal range: 3.4 - 24.1 ng/mL). With these findings, the patient was administered hydrocortisone and levothyroxine, which led to an improvement in her mental status, blood pressure, and electrolyte levels. Further imaging of the pituitary gland and surrounding structures with magnetic resonance imaging was offered to the family but was refused. Considering her challenging prognosis due to clival chondroma and renal cell carcinoma, the patient and her family opted for hospice care. They were subsequently discharged from the hospital to a hospice facility.

## Discussion

Chordomas, derived from embryonic remnants of the notochord, predominantly grow in bone structures and can manifest anywhere along the craniospinal axis. Clival chordomas are rare, affecting <1 per 100,000. The anatomical distribution of chordomas has been documented, with intracranial chordomas accounting for roughly 32%, spinal ones for 32.8%, and sacral ones for 29.2% [5]. The challenge in diagnosing our patient was magnified by the initial presumption that her altered mental state was a direct result of toxic metabolic encephalopathy from an infectious source. The typically asymptomatic nature of chondromas further complicates the diagnostic process. Often, they remain undetected until they trigger significant complications that necessitate medical intervention. In the presented case, it was the patient's altered mental state that initiated her medical consultation.

Panhypopituitarism, which refers to a reduction or complete cessation of pituitary gland hormone production, usually necessitates a comprehensive hormone assessment for diagnosis. In scenarios like the one we encountered, where the chondroma's location is atypical, it becomes even more arduous for healthcare professionals to promptly and correctly diagnose the condition. The relative rarity of clival chondromas further obfuscates their consideration as a differential diagnosis when symptoms indicative of pituitary gland dysfunction emerge. 

An important facet to consider in the context of this case is the broader implications of late diagnoses. Late detection of chondromas, especially in vital regions like the clivus, can lead to severe complications, as evidenced by the resultant panhypopituitarism in our patient. Early identification and intervention might have potentially altered the course of her condition. Furthermore, the relationship between clival chondromas and other systemic diseases, as seen in our patient with concomitant renal cell carcinoma and chronic kidney disease, necessitates further exploration in the literature.

A PubMed search for prior reports of clival chondroma is remarkable for just a handful of cases. For instance, a case documented by Alharbi et al. (2009) illustrates a 50-year-old woman presenting with unilateral hypoglossal nerve palsy as a sign of clival chordoma. This chordoma was identified via CT and MRI imaging, which revealed bone destruction and a mass in the clival region. Surgical intervention followed by radiotherapy was highlighted as a viable treatment approach for managing this clival lesion [6]. A pediatric case by Ghogawala et al. (1991-1992) depicts an eight-year-old female with Ollier’s disease who developed a clival chondroma [7]. Additionally, a case from Japan, as presented by Kikuchi et al. (1979), described a 28-year-old woman with clivus chondroma presenting with muscle weakness and numbness, among other neurological deficits. Partial tumor removal was achieved through a subtemporal approach, highlighting the surgical approach in managing clival chondromas [8]. Meanwhile, a case reported by Tarshis and Briant (1976) discussed a clival chordoma, underscoring the potential for surgical intervention to afford meaningful survival times [9]. These cases collectively illustrate the wide-ranging clinical presentations and the imperative for a multidisciplinary approach to diagnostics and management, particularly when unusual manifestations such as panhypopituitarism are encountered. We therefore believe our discovery to be unique in that, to date, panhypopituitarism secondary to clival chondroma has not been reported.

## Conclusions

This case report underscores the complexity and rarity of chondromas, mainly when they manifest in the clivus, leading to panhypopituitarism. The journey of our 93-year-old female patient, with an extensive medical history, serves as a poignant example of the challenges in diagnosing and managing such a unique medical condition. Chordomas, though generally uncommon, can present in various anatomical locations, with their occurrence in the clivus being infrequent. The initial misattribution of our patient's altered mental status to toxic metabolic encephalopathy from an infectious source illustrates the diagnostic hurdles presented by these typically asymptomatic growths. Furthermore, the late diagnosis of clival chondromas can lead to severe complications, as demonstrated by the emergence of panhypopituitarism in our patient.

The management of panhypopituitarism necessitates a comprehensive evaluation of pituitary hormone levels, and in cases with atypical chondroma locations, the diagnostic process becomes even more intricate. Clival chondromas, given their rarity, are seldom considered a differential diagnosis when symptoms suggestive of pituitary gland dysfunction arise. Our case reminds us of the need for a multidisciplinary approach to diagnostics and management in such exceptional clinical scenarios. While previous reports of clival chondroma are scarce in the literature, each case adds to our understanding of this condition's diverse clinical presentations and potential treatment approaches.
